# Long-Term Evaluation of the Shape of the Reconstructed Diaphragm in Patients with Left-Sided Congenital Diaphragmatic Hernia Using Serial Chest Radiographs and Correlation to Further Complications

**DOI:** 10.3390/jcm13020620

**Published:** 2024-01-22

**Authors:** Christoph von Schrottenberg, Maren Lindacker, Meike Weis, Sylvia Büttner, Thomas Schaible, Michael Boettcher, Lucas M. Wessel, Katrin B. Zahn

**Affiliations:** 1 Department of Pediatric Surgery, University Hospital Mannheim, University of Heidelberg, 68167 Mannheim, Germanykatrin.zahn@umm.de (K.B.Z.); 2 Department of Clinical Radiology and Nuclear Medicine, University Medical Center Mannheim, University of Heidelberg, 68167 Mannheim, Germany; 3 Department of Medical Statistics and Biomathematics, Medical Faculty Mannheim, University of Heidelberg, 68167 Mannheim, Germany; sylvia.buettner@medma.uni-heidelberg.de; 4 Department of Neonatology, University Children’s Hospital Mannheim, University of Heidelberg, 68167 Mannheim, Germany; 5 ERNICA-Center, 68167 Mannheim, Germany

**Keywords:** congenital diaphragmatic hernia, CDH, long-term follow-up, reconstructed diaphragm, chest radiographs, comorbidities, recurrence

## Abstract

*Background*: Defining risk factors for long-term comorbidities in patients after neonatal repair of congenital diaphragmatic hernia (CDH) is an important cornerstone of the implementation of targeted longitudinal follow-up programs. *Methods*: This study systematically assessed serial chest radiographs of 89 patients with left-sided CDH throughout a mean follow-up of 8.2 years. These geometrical variables for the left and right side were recorded: diaphragmatic angle (LDA, RDA), diaphragmatic diameter (LDD, RDD), diaphragmatic height (LDH, RDH), diaphragmatic curvature index (LDCI, RDCI), lower lung diameter (LLLD, RLLD) and thoracic area (LTA, RTA). *Results*: It was demonstrated that the shape of the diaphragm in patients with large defects systematically differs from that of patients with small defects. Characteristically, patients with large defects present with a smaller LDCI (5.1 vs. 8.4, *p* < 0.001) at 6 months of age, which increases over time (11.4 vs. 7.0 at the age of 15.5 years, *p* = 0.727), representing a flattening of the patch and the attached rudimentary diaphragm as the child grows. *Conclusions*: Multiple variables during early follow-up were significantly associated with comorbidities such as recurrence, scoliotic curves of the spine and a reduced thoracic area. Some geometrical variables may serve as surrogate parameters for disease severity, which is associated with long-term comorbidities.

## 1. Introduction

Treating congenital diaphragmatic hernia (CDH), a rare and potentially life-threatening malformation of a newborn’s diaphragm and lungs, does not end with surgical repair and patient discharge. Long-term morbidity in these patients is a well-known problem, especially in those with large defects [1,2]. As survival rates of patients with large defects increase, so does the need for a thorough follow-up accompanying these patients into transition and adulthood [3,4,5,6]. The focus of postdischarge follow-up lies in the detection of recurrences on the one hand and on addressing musculoskeletal (e.g., scoliosis, pectus excavatum), gastrointestinal (e.g., gastroesophageal reflux, small bowel obstruction), pulmonary (e.g., pulmonary hypertension, restrictive and obstructive pulmonary compromise) and neurodevelopmental (e.g., neurodevelopmental delay) sequelae on the other [1,7,8,9]. In 2008, the American Academy of Pediatrics Section on Surgery outlined their proposal of a structured follow-up program for CDH patients [10]. Still, this has not been generally adapted, and follow-up programs differ significantly between high volume centers [9,11]. Robust strategies to assess the long-term risk for developing specific comorbidities (e.g., scoliosis) in CDH patients by defining risk factors beyond the perinatal period remain scarce. In order to facilitate the implementation of standardized long-term follow-up programs and to successfully anticipate and possibly prevent long-term sequelae, the identification of risk factors not only perinatally but continuously throughout long-term follow-up at every visit in the outpatient clinic may be useful.

Numerous studies have defined prenatal risk factors, such as total fetal lung volume (TFLV), observed-to-expected lung-to-head ratio (o/e LHR) or intrathoracically herniated liver, as predictive factors for the need of extracorporeal membrane oxygenation (ECMO), as well as for survival and (short-term) morbidity [12,13,14,15,16,17,18,19]. Perinatal parameters such as the CDH study group predictive survival score, initial blood gas variables, the Brindle score, the McGoon index, the 12–24 h Score for Neonatal Acute Physiology, Version II (SNAP-II), the need for ECMO, the use of inhaled nitric oxide (iNO) or defect size, which often correlate with each other, have been found to be associated with survival and short-term morbidity (e.g., duration of ventilation, chylothorax, hemorrhage, length of hospital stay) [19,20,21,22]. Multiple studies have reported incidences of long-term (surgical) morbidity in CDH patients, but only a few have identified reliable risk factors such as open surgery, patch repair and ECMO—all surrogate parameters for large defects—to predict these long-term morbidities [23,24,25,26]. Recently, Weis et al. demonstrated that the chest radiographic thoracic area (CRTA), measured on chest radiographs performed postnatally, can serve as a good prognostic parameter for morbidity and mortality, even superior to the prenatally assessed o/e LHR [27]. Another study by Dassios et al. also proposed the CRTA as a sensitive predictor for survival to discharge in neonates with CDH [28].

**Figure 1:**
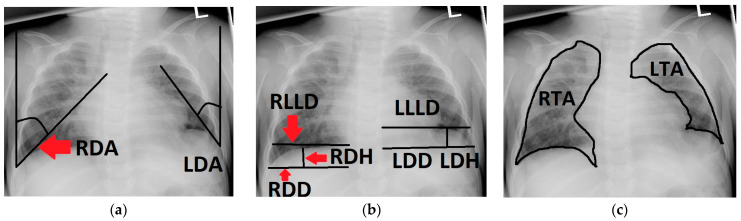
Chest radiograph of a 6-month-old patient after left-sided CDH repair with a cone-shaped patch in the neonatal period; measurements of the following variables are outlined (black line) and labeled on the patient’s right side (red arrows): (**a**) right and left diaphragmatic angle (RDA, LDA); (**b**) right and left diaphragmatic diameter (RDD, LDD), right and left diaphragmatic height (RDH, LDH) and right and left lower lung diameter (RLLD, LLLD); (**c**) right and left thoracic area (RTA, LTA).

Even though respiratory muscles and the diaphragm in particular are of major importance for lung development, the literature on the shape and function of the reconstructed diaphragm during infancy and childhood in patients with congenital diaphragmatic hernia is scarce [29]. So far, no studies have assessed the radiologic morphology or the geometrical proportions of the reconstructed diaphragm in CDH patients during follow-up. Short et al. examined the curvature of the diaphragm and its rib insertion level on postoperative chest radiographs in 127 CDH patients with a patch rate of 46%. They hypothesized that a postoperatively flat diaphragm was associated with a higher recurrence rate during follow-up. They created a new parameter called the “diaphragmatic curvature index” (DCI), which was described via the quotient of the diaphragmatic diameter and the diaphragmatic height, indicating its curvature (see Figure 1), and which they considered a surrogate parameter for the tension, under which the diaphragm had been reconstructed. They concluded that there was no significant difference of the DCI between patients with and without recurrence in either the group with or in the group without patch repair [30]. Short et al. only analyzed radiographs taken after CDH repair in the neonatal period, and no other comorbidities such as scoliosis were assessed during follow-up. For the first time, our study gives a systematic evaluation of the shape of the reconstructed diaphragm and the thorax over a follow-up time of a mean of 8.2 years in 89 patients with left-sided CDH after both primary and patch repair. Further, by analyzing several geometric variables of the reconstructed diaphragm and the patients’ thorax, an attempt was made to identify radiologic risk factors for the development of the following comorbidities during the course of long-term follow-up: recurrence, scoliosis and reduced thoracic area/pulmonary hypoplasia.

## 2. Materials and Methods

After gaining approval by the local ethics committee (2018-592N-MA), a review of all CDH patients born at or admitted to our institution between January 1998 and December 2005 was performed.

We retrospectively analyzed patient data and baseline characteristics acquired throughout our standardized prospective long-term follow-up of CDH patients, as previously described by our group [25]. Chest radiographs taken in supine position when necessary and in upright position when possible were analyzed by two study group members under the supervision of an attending radiologist specializing in pediatric radiology. The following variables were measured in each radiograph using SyngoShare (Siemens AG, Medical Solutions, Forchheim, Germany, www.siemens.com/syngo, accessed on 1 December 2022):

- The left and right diaphragmatic angle (**LDA**, **RDA**), which were defined by the angle between the lateral chest wall and the tangent to the convex side of the ipsilateral diaphragm coming from the costodiaphragmatic recessus.
- The left and right diaphragmatic diameter (**LDD**, **RDD**), measuring from the costodiaphragmatic recessus to the medial limit of the diaphragm.
- The left and right diaphragmatic height (**LDH**, **RDH**), measured as the perpendicular line from the diaphragmatic diameter to the apex of the diaphragm.
- The left and right lower lung diameter (**LLLD**, **RLLD**), measuring the width of the lung from its limit at the lateral chest wall to its medial limit at the level of the apex of the diaphragm.
- The left and right thoracic area (**LTA**, **RTA**) were defined by delineating the outer border of the lung tissue, excluding the mediastinum and the cardiac shadow.
- The total thoracic area (**TTA**) was calculated via the sum of **LTA** and **RTA**.
- The left and right diaphragmatic curvature index (**LDCI**, **RDCI**) were calculated via the quotient of the diaphragmatic diameter and the diaphragmatic height (e.g., **LDD**/**LDH** = **LDCI**). A large **LDCI** therefore indicates a flat diaphragm with only a small curvature.

**Figure 2:**
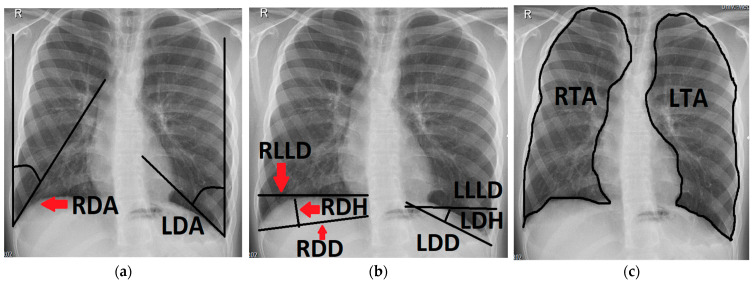
Chest radiograph of a 16-year-old patient after left-sided CDH repair with a cone-shaped patch in the neonatal period; measurements of the following variables are outlined (black line) and labeled on the patient’s right side (red arrows): (**a**) right and left diaphragmatic angle (RDA, LDA); (**b**) right and left diaphragmatic diameter (RDD, LDD), right and left diaphragmatic height (RDH, LDH) and right and left lower lung diameter (RLLD, LLLD); (**c**) right and left thoracic area (RTA, LTA).

Figure 1 and Figure 2 illustrate the measuring. These variables were measured at the following time points during follow-up: 6 ± 2 months, 12 ± 2 months, 24 ± 4 months, 3.5 ± 0.5 years, 6 ± 1 years, 10 ± 2 years and 15.5 ± 2.5 years. As a control group, chest radiographs of 70 age-adjusted patients with non-pulmonary and non-musculoskeletal diseases (e.g., acute lymphatic leukemia) whose chest radiographs were taken after operative implantation of a central venous catheter or for other reasons were analyzed in the same way. In order to identify possible risk factors for developing the comorbidities recurrence, scoliosis and reduced thoracic area/lung hypoplasia in association with the configuration of the reconstructed diaphragm, the acquired variables were then compared between patients that presented with the respective comorbidity and those that did not. In statistical analysis, continuous variables were reported as medians or means and compared using one-way analysis of variance (ANOVA) where applicable or 2-sample independent *t*-tests or Mann–Whitney *u*-tests (non-normal data). Significant variables were then analyzed using logistic regression in order to identify independent predictors. Contingency tables were analyzed with Fisher’s exact test or chi-square test where applicable. *p*-values < 0.05 were considered significant.

## 3. Results

### 3.1. Patient Characteristics

Of 226 newborns with the diagnosis of CDH born at or admitted to our department between January 1998 and December 2005, a total of 137 (60.6%; 59 female, 78 male) patients had to be excluded from this study. In summary, 43 (19%) patients were excluded due to right-sided hernia, 1 patient had a bilateral hernia, and 45 (19.9%) patients died after an average of 30.8 days and were thus not available for long-term follow-up. No evaluable radiologic data or complete loss to follow-up were the reasons for exclusion with another 47 (20.8%) patients. One patient was excluded because he was operated on elsewhere with the implantation of a resolvable patch.

Thus, 89 patients (50 female, 39 male) with left-sided CDH were eligible for measurements on serial chest radiographs. At the time of surgery, the CDHSG classification system for defect size did not yet exist [6]. Therefore, for this study, patients were divided according to the surgical method of diaphragmatic reconstruction: defects that could be primarily closed were categorized as small, and defects that required the implantation of a patch were categorized as large defects. Accordingly, 35 patients (39%) had a small defect and 54 (61%) patients had a large defect. In all patients, access to the diaphragmatic defect was gained through a median laparotomy. To avoid closure under tension, a cone-shaped polytetrafluoroethylene patch was used for diaphragmatic reconstruction in CDH patients with large defects [31]. Venoarterial ECMO (VA-ECMO) therapy was required in 31 patients (35%), and surgical repair of the diaphragmatic defect was usually performed one or two days after decannulation. VA-ECMO was required significantly more often in patients with large defects (55.6 vs. 2.9%, *p* < 0.0001) and lasted 7.6 days on average. Birth weight (2791 vs. 3124 g, *p* = 0.009) and length (50.0 vs. 51.5 cm, *p* = 0.005) were significantly lower in patients with large defects who were also born at an earlier gestational age (37 + 1 vs. 38 + 0 weeks of gestational age, *p* = 0.003). All nine detected recurrences (10%) appeared in patients with large defects at an average age of 13 months (interquartile range: 12 months, 183 months). One re-recurrence was detected at the age of 10 years. Mean follow-up time was 8.2 years (range: 5 months–18 years). Patients with large defects were eligible for follow-up significantly longer than patients with small defects (9.1 vs. 6.9 years, *p* = 0.04). Table 1 gives an overview of patients’ characteristics.

### 3.2. Geometrical Variables

**Figure 3:**
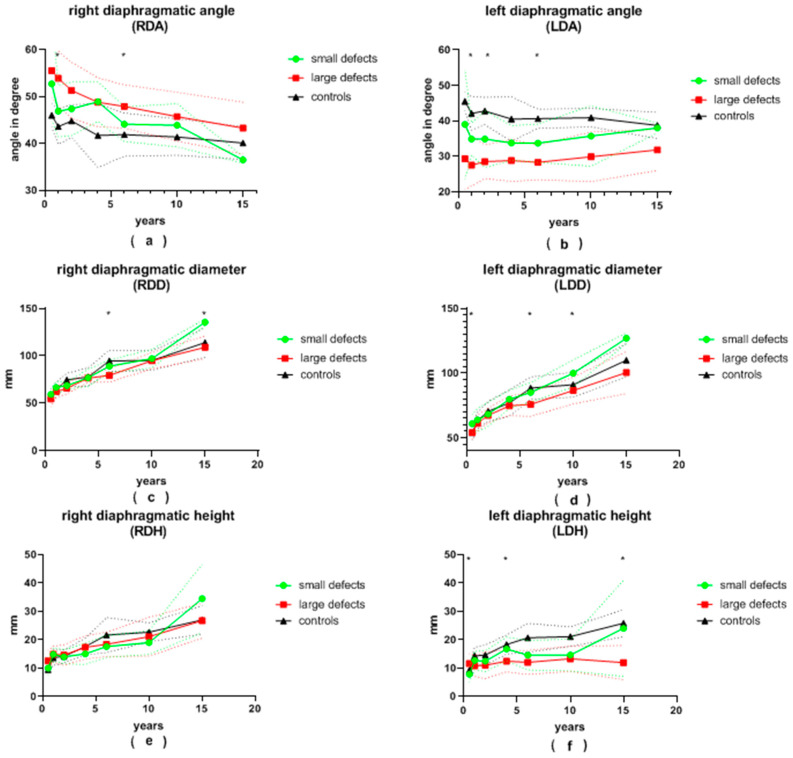
(**a**) right diaphragmatic angle (RDA), (**b**) left diaphragmatic angle (LDA), (**c**) right diaphragmatic diameter (RDD), (**d**) left diaphragmatic diameter (LDD), (**e**) right diaphragmatic height (RDH) and (**f**) left diaphragmatic height (LDH) in patients with small and large congenital diaphragmatic hernia defects at the ages of 6 ± 2 months, 12 ± 2 months, 24 ± 4 months, 3.5 ± 0.5 years, 6 ± 1 years, 10 ± 2 years and 15.5 ± 2.5 years during long-term follow-up; continuous lines represent mean values; dotted lines represent standard deviation; *, significant differences between patients with small and large defects are marked with an asterisk.

**Figure 4:**
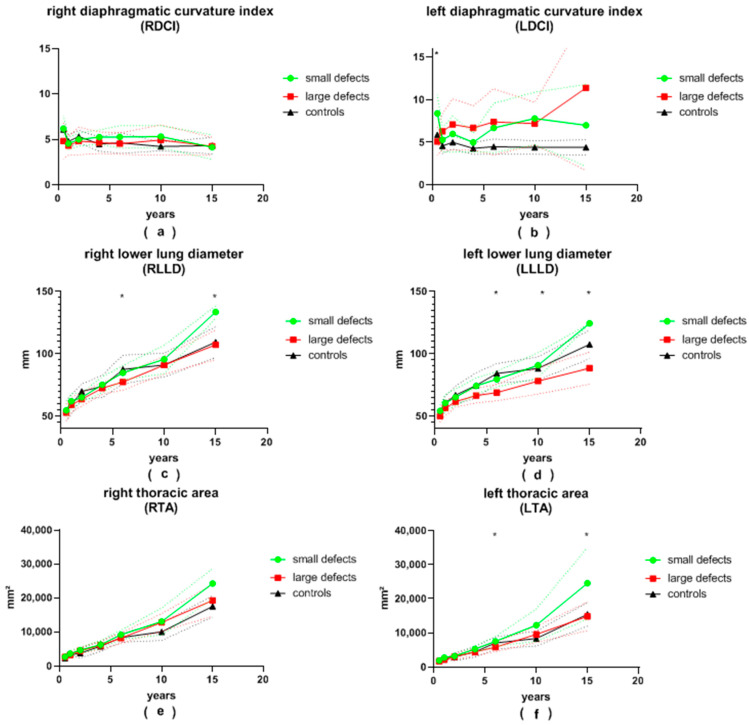
(**a**) right diaphragmatic curvature index (RDCI), (**b**) left diaphragmatic curvature index (LDCI), (**c**) right lower lung diameter (RLLD), (**d**) left lower lung diameter (LLLD), (**e**) right thoracic area (RTA) and (**f**) left thoracic area (LTA) in patients with small and large congenital diaphragmatic hernia defects at the ages of 6 ± 2 months, 12 ± 2 months, 24 ± 4 months, 3.5 ± 0.5 years, 6 ± 1 years, 10 ± 2 years and 15.5 ± 2.5 years during long-term follow-up; continuous lines represent mean values; dotted lines represent standard deviation; *, significant differences between patients with small and large defects are marked with an asterisk.

A total of 209 chest radiographs of 54 patients after patch repair and 84 chest radiographs of 35 patients after primary repair were analyzed. Additionally, 70 chest radiographs of age-adjusted patients admitted for other reasons and without CDH were analyzed and served as the control group. All variables are visualized in Figure 3 and Figure 4, and data are displayed in detail in Table S1 of the Supplementary Material.

#### 3.2.1. Ipsilateral Left Side

The **LDA** of patients with large defects was generally more pointed than that of patients with small defects. This difference was significant at the ages of 12 and 24 months, as well as at the age of 6 years, and almost reached significance at the age of 6 months and 10 years (28 vs. 35°, *p* = 0.008; 28 vs. 35°, *p* = 0.002; 28 vs. 34°, *p* = 0.001; 28 vs. 36°, *p* = 0.056 and 30 vs. 36°, *p* = 0.075, respectively). At the age of 6 months, as well as at 6 and 10 years of age, patients with small defects presented with a significantly larger **LDD** than patients with large defects (61 vs. 54 mm, *p* = 0.018; 85 vs. 76 mm, *p* = 0.001; 100 vs. 87 mm, *p* = 0.004, respectively). Initially, the average **LDH** was significantly greater in patients with large defects (12 vs. 8 mm, *p* = 0.01 at 6 months). It became significantly smaller than that of patients with small defects during follow-up (12 vs. 17 mm, *p* = 0.036; 12 vs. 24 mm, *p* = 0.045 at 3.5 and 15.5 years of age, respectively). After the age of 6 months, the **LDCI** remained stable at around 4.7 in the control group. At the age of 6 months, the **LDCI** in patients with small defects was significantly higher in comparison to patients with large defects (8.4 vs. 5.1, *p* < 0.001). Afterwards, the **LDCI** was constantly higher in patients with both large and small defects compared to the control group, but significance was reached only for patients with large defects in comparison to the control group. The **LLLD** was significantly smaller in patients with large defects compared to patients with small defects at the age of 6 years and older (69 vs. 80 mm, *p* < 0.001; 78 vs. 91 mm, *p* = 0.007; 89 vs. 125 mm, *p* = 0.001) and almost reached significance at the 3.5-years visit (67 vs. 74 mm, *p* = 0.057). In children older than 2 years, the **LLLD** was also smaller in patients with large defects in comparison to the control group (67 vs. 75 mm, *p* = 0.019; 69 vs. 84 mm, *p* < 0.01; 78 vs. 88 mm, *p* = 0.027; 89 vs. 108 mm, *p* = 0.002). On the other hand, patients with small defects did not differ significantly from the control group. The average **LTA** was always smaller in the group of patients with large defects compared to the group of patients with small defects. This reached significance at the ages of 6 and 15.5 years (5855 vs. 7552 mm^2^, *p* < 0.001; 14,836 vs. 24,534 mm^2^, *p* = 0.015) and nearly reached significance at the age of 10 years (9572 vs. 12,294 mm^2^, *p* = 0.05). At the age of 10 and 15.5 years, the **LTA** of patients with small defects was greater than in the control group (12,294 vs. 8388 mm^2^, *p* = 0.015; 24,534 vs. 15,464 mm^2^, *p* = 0.031).

#### 3.2.2. Contralateral Right Side

The average **RDA** was less pointed in all patients with CDH compared to the control group. Patients with large defects presented with a more obtuse angle than patients with small defects at every follow-up visit. This was significant at 12 months and 6 years of age (54 vs. 47°, *p* = 0.001; 48 vs. 44°, *p* = 0.009). The mean **RDD** was always smaller in patients with large defects than in patients with small defects, yet statistical significance was reached only at 6 and 15.5 years of age (79 vs. 89 mm, *p* < 0.001; 109 vs. 136 mm, *p* = 0.029). The average **RDH** and **RDCI** were both similar between patients with small and large defects during the entire follow-up. The **RLLD** tended to be smaller in patients with large defects compared to both the control group and to patients with small defects. Significance between the two groups of CDH patients was reached at 6 and 15.5 years of age (77 vs. 85 mm, *p* = 0.002; 107 vs. 134 mm, *p* = 0.017). Similar to the **LTA**, the **RTA** was consistently smaller in patients with large defects compared to patients with small defects. Patients with small defects presented with a larger **RTA** than the control group, but no statistical significance was reached. At the age of 10 years, the **RTA** was significantly larger in patients after patch repair compared to the control group (12,820 vs. 10,051 mm^2^, *p* = 0.034).

### 3.3. Clinical Data

#### 3.3.1. Recurrence

Recurrence occurred in 9/89 patients (10.1%), solely after initial patch repair. Median age at detection and repair of recurrence was 13 months (interquartile range: 12–183 months). All previously described measurements were compared at the different intervals of follow-up between patients that developed a recurrence and those that did not. In patients with recurrence, variables measured after the repair of the recurrence were not included, which led to a distinct reduction in available data for these patients. All data are displayed in Table S2 of the Supplementary Material.

At the age of 6 months, the **LDCI** was significantly smaller in patients with recurrence than in patients without recurrence (3.9 vs. 6.5, *p* = 0.045). At the age of 12 months as well as at 10 years, no variable showed any significant difference between the two groups. At 24 months of follow-up, only the **RDH** was significantly greater in patients with recurrence than in patients without (19 vs. 14 mm, *p* = 0.01). At the age of 3.5 years, **TTA** was the only variable to present a significant difference between the two groups (6673 vs. 10,845 mm^2^, *p* = 0.037). At 6 years, the following variables showed significant differences between patients with and without recurrence, respectively: **RDD** (70 vs. 83 mm, *p* = 0.022), **RLLD** (66 vs. 81 mm, *p* = 0.005), **LTA** (3669 vs. 6593 mm^2^, *p* = 0.009), **RTA** (6065 vs. 8636 mm^2^, *p* = 0.018) and **TTA** (9734 vs. 15,050 mm^2^, *p* = 0.015).

In multivariable logistic regression analysis, none of the variables could be identified as significant independent predictors for recurrence.

#### 3.3.2. Curvature of the Spine/Scoliosis

A curvature of the spine was identified in 31.6% of all patients (18/57) with a follow-up of at least eight years (mean: 11.1 years, range: 8–18 years). Within this group, 47% of patients with large defects (15/32) and 12% of patients with small defects (3/25) developed a curvature of the spine (OR = 6.47 [95% CI 1.43–11.81], *p* = 0.0088). In total, 56% of these patients (10/18) had received ECMO therapy, as opposed to only 26% of patients (10/39) without curvature of the spine (OR = 3.63 [95% CI 1.06–11.41], *p* = 0.0386). Altogether, 26 scoliotic curves were identified with a mean Cobb angle of 15° (range 9°–25°). Two patients had a Cobb angle < 10°, while two patients (4%) had a Cobb angle of >20° and thus a scoliosis requiring treatment. Ten patients had a single curve and eight had a double curve. A total of 55% of the cranial curves were convex to the patients’ right side, away from the defect side. Of the eight patients with a double curve, seven had large defects and five patients presented with the cranial curve convex to the left side.

All acquired variables that were gathered at the ages of 6, 12 and 24 months and 3.5 and 6 years were compared between patients with and without scoliosis. All data are displayed in Table S3 of the Supplementary Material. In general, the diaphragmatic angle—irrespective of side—and most of the right-sided values (**RDD**, **RDH**, **RDCI**, **RTA**) did not show a significant difference at any point during follow-up. For each of the following variables, a significant difference between the two groups was noted at different times during follow-up: the **LDD** at the age of 6 months was smaller in patients with scoliosis (51 vs. 59 mm, *p* = 0.011). The **LDH** at the age of 3.5 years was smaller in patients with scoliosis (11 vs. 15 mm, *p* = 0.01). The **LDCI** at the age of 3.5 years was larger in patients with scoliosis, as the curvature of the reconstructed diaphragm was reduced in these patients (7.9 vs. 5.3, *p* = 0.045). The **LLLD** at the age of 6 months was smaller in patients with scoliosis (46 vs. 54 mm, *p* = 0.004). At the age of 6 years, the **LTA** and the **TTA** were accordingly smaller (5696 vs. 7077 mm^2^, *p* = 0.0047; 13,226 vs. 15,960 mm^2^, *p* = 0.004). At the age of 3.5 years, the **RLLD** was smaller in patients with scoliosis (72 vs. 76 mm, *p* = 0.049).

In multivariable logistic regression analysis, none of the variables could be identified as significant independent predictors for a scoliotic curve of the spine.

#### 3.3.3. Total Thoracic Area

The median **TTA** of the control group at the age of 10 years was 20,025 mm^2^. Data of 36 CDH patients were available at 10 years of age. The cohort of CDH patients was then divided into two groups, those that presented with a **TTA** smaller than the median of the control group (n = 10) at the age of 10 years and those with a larger **TTA** (n = 26), and variables assessed during follow-up before the age of 8 years were compared. At 6 months of age, the **RDD**, the **LDH** and the **RLLD** were all significantly smaller in the group of patients that later presented with a reduced **TTA** (49 vs. 61 mm, *p* = 0.003; 8 vs. 11 mm, *p* = 0.02 and 48 vs. 57 mm, *p* = 0.004, respectively). At 12 months of age, the **RDA** was significantly bigger in the group that developed a reduced **TTA** (59 vs. 50°, *p* = 0.014). At 24 months of age, the **LDD**, the **LLLD** and the **RLLD** were significantly smaller in the reduced **TTA** group (62 vs. 68 mm, *p* = 0.038; 58 vs. 63 mm, *p* = 0.043 and 60 vs. 66 mm, *p* = 0.0498, respectively). At 3.5 years, as well as at 6 years of age, no significant difference in any of the measured variables between the two groups could be detected. All data are displayed in Table S4 of the Supplementary Material.

In multivariable logistic regression analysis, none of the variables could be identified as a significant independent predictor for a reduced thoracic area/lung hypoplasia.

## 4. Discussion

### 4.1. Geometric Variables

This study is the first to systematically assess different radiologic variables of the diaphragm, lower lung diameter and thoracic area on the ispi- and contralateral side in patients with left-sided CDH on serial chest radiographs over a mean follow-up time of more than 8 years. In general, a reconstructed diaphragm and the ipsilateral thorax differ in some aspects from the contralateral right side and also from the control group. Furthermore, the reconstructed diaphragm of CDH patients with large defects requiring patch implantation differs from that of CDH patients with primary repair. In particular, it presents with an increased curvature during infancy and assimilates during growth, which is best described using the diaphragmatic curvature index (**DCI**).

To date, one other study has described the **DCI** before, but it only evaluated it immediately postoperatively in CDH patients, whereas our study gives insight into the dynamic formation of the shape of the diaphragm over time [30].

#### 4.1.1. Ipsilateral Left Side

Patients with large defects present with a more pointed **LDA**. The reduced **LDD** in patients with large defects at 6 months and 6 and 10 years of age compared to patients with small defects is in line with their reduced **LTA** at 6 and 15.5 years. These differences may be considered a consequence of an asymmetric and reduced growth of the left thoracic cavity and may serve as a surrogate parameter for more severe lung hypoplasia. This is further supported by the significantly reduced **LLLD** in patients older than 3.5 years compared to patients with small defects. Furthermore, an enlarging difference of the **LLLD** could be noted during growth, which seems to reflect the persistence of lung hypoplasia and its reduced catch-up growth capacity. Interestingly, the **LLLD** of patients with small defects did not differ from that of the control group, thus excluding persistent lung hypoplasia in these patients. In contrast, in patients with large defects 3.5 years and older, the **LLLD** was constantly smaller than in the control group, which may also reflect their persistent lung hypoplasia. The **LDH** initially was greater in patients with large defects. This might be due to the implantation of a cone-shaped patch in our cohort, which is designed to imitate the convex contour of the diaphragm and enlarge the hypoplastic abdominal cavity in order to reduce the need for the implantation of an abdominal wall patch. On the other hand, redundant thoracic volume is reduced in patients with more severe lung hypoplasia [31]. During follow-up, the **LDH** of patients with small defects and the control group increase continuously, depicting physiologic growth of a diaphragm preserving its curved shape. In contrast, the height of the left-sided diaphragm of CDH patients with large defects rather seems to stagnate, indicating the inability to grow into its natural shape. It can be noted that the cone-shaped patch rather flattens over time by being stretched through the pulling force of the circumferentially attached rudimentary diaphragm and growing rib cage. The same effect is reflected by the **LDCI**. Particularly in adolescence, the **LDCI** was significantly higher in patients with large defects than in the control group, indicating a less convex-shaped and rather flattened diaphragm in these patients. In our cohort, the **LTA** was consistently smaller in patients with large defects than in patients with small defects, which seems reasonable, as these patients suffer from more severe lung hypoplasia, yet statistical significance was reached only at the ages of 6 and 10 years. The **LTA** being larger in patients with small defects than in the control group at the ages of 10 and 15.5 years may be a sign of an overexpansion of the ipsilateral lung due to obstructive lung function compromise.

#### 4.1.2. Contralateral Right Side

A more obtuse **RDA** was detected in patients with large defects. The **RDD** was generally smaller in patients with large defects than in patients with small defects and in the control group, yet statistical significance was reached only sporadically. Findings were similar for the **RLLD**. This may reflect that patients with large CDH suffer from lung hypoplasia not only on the ipsi- but also on the contralateral side, which thus also seems to persist during growth. The average **RTA** of patients with small defects was always larger than that of the control group, but no statistical significance was reached. At the age of 10 years, the **RTA** of patients with large defects was larger than in the control group. These findings may theoretically indicate a compensatory growth of the non-affected lung, leading to a stronger growth of the right-sided thoracic cavity in these patients. Also, an overexpansion of the contralateral lung due to obstructive lung function compromise could be another reason. The **RTA** generally being smaller in patients with large defects than in patients with small defects may be due to a generally more severe lung hypoplasia in these patients, but not only on the affected side.

Results from Stoll-Dannenhauer et al., who analyzed CT scans, show that lung volume in CDH patients at the age of 4.5 years did not differ from that of controls on either the ipsi- or the contralateral side [32]. Still, compensatory (catch-up) growth of the ipsi- and contralateral lung and thorax in CDH patients remains a controversial topic in the literature. Performing sequential pre- and postnatal MRIs in CDH patients, Schopper et al. could demonstrate that during the postnatal period, the contralateral lung grew faster than the ipsilateral lung in patients with small defects. In patients with large defects, the compensatory growth of the ipsilateral lung surpassed that of the contralateral side [33]. Contrarily to Schopper et al., we found an increase in both the **LTA** and **RTA** that continued equally in both groups, and in adolescence, an even stronger increase in **LTA** and **RTA** in patients with small defects could be noted [33]. Nagaya et al. were also able to demonstrate lung growth in CDH patients assessing lung volume using CT scans. Interestingly, their results show that patients suffering from severe respiratory distress at admittance showed a notable growth in the ipsilateral lung but lacked adequate growth of arterial vessels, thus leading to a significant decrease in mean perfusion, thus leading to a significant decrease in the ratio of mean perfusion to volume from 87% to 67%.” [34]. Furthermore, several MRI studies have shown a persistent lung hypoplasia, reduced density and lung perfusion at older age [35,36,37,38,39].

### 4.2. Clinical Data

#### 4.2.1. Recurrence

The recurrence rate of 17% (9/54) in our group of patients with patch repair is similar to the average recurrence rate of 16.2% reported in a systematic review and meta-analysis by Heiwegen et al. [26]. No recurrence occurred in our group of patients with primary repair, whereas Heiwegen et al. reported a recurrence rate of 5.8%. Defect size is reported to be a risk factor for recurrence and morbidity by multiple authors [25,26].

Analyzing the different geometrical variables on serial chest radiographs concerning the incidence of recurrence, some variables showed significant differences at a few time periods despite the small number of patients with recurrence. The **TTA** was significantly reduced at the ages of 3.5, 6 and 15.5 years in patients that suffered from a recurrence compared to patients without recurrence. The **TTA** may be considered a surrogate parameter for a reduced thoracic cavity as a sign of severe pulmonary hypoplasia, which in turn is more distinct in patients with large defects—the group of patients more prone to developing a recurrence.

Short et al. hypothesized that closure of the diaphragmatic defect under tension would facilitate recurrence and that this could best be described by a high **LDCI**. In their study, they measured a **LDCI** of 6.00 and 6.46 in patients with and without recurrence, respectively (*p* = 0.853), falling short of statistically confirming their hypothesis [30]. In contrast, patients with recurrences in our study showed a significantly reduced **LDCI** at the age of 6 months (3.9 vs. 6.5, *p* = 0.045). This could be due to the fact that implanted cone-shaped patches overcompensate the physiologic shape of the diaphragm during the first months after the operation. As patch repair was only performed in patients with large defects and these are prone to having recurrences, it seems obvious why patients with recurrences tend to have a reduced **LDCI** in the first months of life. In accordance with this assumption, these patients presented a **LDCI** slightly larger than patients without recurrence during later follow-up, as the patch has flattened markedly by this age, and a curved shape of the diaphragm often cannot be preserved into childhood and adolescence in patients with large defects and a hypoplastic diaphragmatic rim. Similarly, in patients with recurrence, the **LDH** was reduced after the age of two years, which expresses the flattened diaphragm as well. Due to the small number of patients, this did not reach statistical significance. Furthermore, at the age of six years, several variables were all significantly smaller in patients with recurrences than in patients without: **RDD**, **RLLD**, **LTA**, **RTA** and **TTA**. As these variables may be surrogate parameters for defect size, patients with large defects suffer from bilateral lung hypoplasia and thus a reduced thoracic growth with a subsequently also reduced diameter of the contralateral diaphragm and lower lung.

#### 4.2.2. Curvature of the Spine/Scoliosis

In our study, the prevalence of curvature of the spine within the group of patients available for a follow-up of at least 8 years was 31.6% (18/57); a significant scoliosis with a Cobb angle > 20° was observed in two patients (4%). Kuklova et al. reported a prevalence of scoliosis of 26% in their radiographic follow-up of 53 patients but also reported that only 5% presented with a clinically significant scoliosis. Surprisingly, in their cohort, there was no significant difference in the prevalence of scoliosis between patients with small and large defects (10/43 patients vs. 4/10 patients, *p* = 0.426). In our cohort, the prevalence of radiologic detection of a curvature of the spine was significantly higher in patients with large defects (47 vs. 12%, OR 6.47, 95% CI 1.43–11.81; *p* = 0.0049). It needs to be considered that in their cohort, the reported patch rate was markedly lower than in our cohort (19 vs. 61%, *p* = 0.0001). Furthermore, significantly more patients with scoliosis had received ECMO in our cohort (55.6 vs. 25.6%, *p* = 0.0386). Thus, more severe CDH patients are included in our study, but this does not seem to affect the overall rate of scoliosis negatively. One more difference between the two study cohorts is the operative approach: in Kuklova’s cohort, a transverse laparotomy was used, whereas we performed a median laparotomy in all patients [40]. The muscle sparing abdominal approach may thus lead to a reduced rate of scoliosis and explain why the rate of scoliosis after primary diaphragmatic reconstruction was almost twice as high in Kuklova’s cohort (10/43 [23%] vs. 3/25 [12%]). In 1996, Vanamo et al. published a long-term follow-up study of 60 patients with CDH/eventration after neonatal surgery and analyzed them at a mean age of 29.6 years. They reported a prevalence of clinically significant scoliosis (Cobb angle ≥ 10°) in 27% (16/60) of patients [41]. Additionally, 31 patients (52%) presented with a clinically inapparent scoliotic curve with a Cobb angle < 10° on radiographs. They reported twelve patients (20%) with a Cobb angle of 10–24°, two patients (3%) with a Cobb angle of 25–39° and two patients (3%) with a Cobb angle of >40°. In our cohort, two patients (4%) with mild curvature of the spine had a Cobb angle of less than 10°, fourteen patients (23%) had a Cobb angle of 10–20° and two patients (4%) had a Cobb angle > 20°. Taking into account the younger age group in our cohort and the much higher rate and severity of scoliosis at older age, scoliosis of the spine should be distinctly looked for, and treatment ought to be initiated during childhood in order to prevent worsening and severe scoliosis at older age. This is particularly supported by recently published data by Kraemer et al., who performed a long-term follow-up of 68 adults after neonatal CDH repair at an average age of 23 years that reported a prevalence of 42.6% of scoliosis in chest CT scans [42]. In the literature, idiopathic adolescent scoliosis (IAS) affects 1–4% of adolescents, is more common in females and appears to have a distinct distribution of right to left-sided thoracic scoliosis of 9:1 [43,44]. Vanamo et al. described a gender distribution (male: female) of 6:10 in their cohort, and in ours it was 4:14 (*p* = 0.0634). In contrast to the etiology of IAS, Vanamo et al. demonstrated a slightly predominant distribution of left to right-sided thoracic scoliotic curves of 10:6 in their CDH cohort, which is in contrast to our data, with a fairly even distribution of 10:8. We could not detect a significant difference between the Cobb angle of patients according to initial defect size (11° in patients with small defects vs. 14° in patients with large defects, *p* = 0.252). The fact that seven of the eight patients with double curves had large defects and five presented with the cranial curve convex to the left side may indicate that large diaphragmatic defects lead to more severe scoliosis. Vanamo et al. reported that “the mean curve [of the scoliosis] was significantly greater in patients who had a large defect (*p* < 0.05)”, but no other risk factors were identified [41]. In our study, some variables showed significant differences between patients with and without scoliosis at scattered time points. Yet, no consistency could be noted other than if these variables were taken as surrogate parameters for defect size, they could be inconsistently associated with the presence of scoliosis at the age of 8 years and older. Just like Vanamo et al., we hypothesize that hypoplasia of the ipsilateral lung leads to a reduced growth of the ipsilateral hemithorax (thoracic asymmetry), which in turn may promote scoliosis, as was described in a biomechanical model by Stokes et al. [45]. Still, the etiology of scoliosis in CDH patients is not fully understood and is most likely multifactorial.

#### 4.2.3. Total Thoracic Area

CDH patients that at the age of 10 years presented with a **TTA** smaller than the control group’s median had a significantly smaller **RDD** and **RLLD** in infancy, which could be an indicator for contralateral lung hypoplasia. The **RDA** was the only parameter at the age of one year to be significantly larger in the group with a reduced **TTA**. As patients with large defects present with a more obtuse **RDA** during infancy, as visualized in Figure 1, the result that these patients as “teens” present with a reduced **TTA** again supports the idea of geometrical variables serving as indicative parameters for disease severity. This idea is reinforced by the fact that patients with a reduced **TTA** also presented with a significantly reduced **LDD**, **LLLD** and **RLLD** at the age of 24 months.

## 5. Limitations and Strengths

A large number of patients had to be excluded from this study due to missing radiographs or due to radiographs that were not applicable for geometrical evaluation. Exclusion of right-sided CDH reduced our cohort even more. On the other side, it contributed to unifying the cohort. Significant results in sub-analysis of right-sided CDH would have been difficult to achieve due to the small number of patients. Specific characteristics on the shape of the reconstructed diaphragm in this particular patient group remain unknown and should be of interest for further studies. In general, data were quite inconsistently available, with some patients having taken part in every outpatient clinic visit and others only having taken part sporadically. Complete loss to follow-up in our cohort of patients with left-sided CDH was 21%. In a report of a multicenter study in Japan by Yamoto et al., 626 CDH survivors were followed up with at 1.5, 3, 6 and 12 years of age, with a melting participation rate of 100%, 82.9%, 42.2% and 11.7%, respectively, as patients grew older [46]. Therefore, long-term follow-up data are difficult to obtain.

Another limitation of this study is that chest radiographs only give a two-dimensional image of the diaphragm and the thorax, making measurements quite imprecise. Three-dimensional measuring techniques give superior insight into the morphology, yet feasibility needs to be considered. Further, scoliosis was assessed exclusively based on radiographs, so no certain statement can be made on whether some of the discrete radiologic findings that were included in this study had any clinical relevance. Due to the sole use of cone-shaped patches in larger defects, no analysis concerning different implantation techniques of patches could be performed. The small number of patients with recurrence made analysis at different time points difficult. This may also be the reason why no independent risk factors could be identified in multiple regression analysis.

The strength of this study is that it analyzes a large single-center cohort with a rather uniform surgical technique. It is the first long-term follow-up with serial chest radiographs and longitudinal measurements and may offer new aspects to better define risk factors for long-term complications.

## 6. Conclusions

To our knowledge, this is the first study to systematically assess the shape of the reconstructed diaphragm in CDH patients over a mean follow-up time of more than 8 years. It was demonstrated that the reconstructed diaphragm differs in its shape from the unaffected side, as well as from unaffected patients. After patch implantation, there seems to be a characteristic switch from a small DCI at younger age to a large DCI at older age. For the first time, an objectively measurable variable can be presented that demonstrates the effect of child growth on the reconstructed diaphragm being stretched flat despite implantation of a cone-shaped patch. Variables that could be regarded as surrogate parameters for defect size correlated with the detection of scoliosis or recurrence. The high rate of radiologically apparent curvatures of the spine emphasizes the need for standardized, interdisciplinary postdischarge long-term follow-up programs in high volume centers in order to better recognize, monitor and treat long-term sequelae in these patients. Furthermore, the enlarging difference in measurements between patients with small and large defects seems to reflect a lack of catch-up growth and more severe lung hypoplasia that persists beyond neonatal age and infancy. Our study could not identify specific risk factors when analyzing chest radiographs over a long time period but rather depicted sporadic correlations between defect size and morbidity in CDH patients. It may serve other study groups and clinicians as a first approach to systematically analyze the shape of the reconstructed diaphragm over the course of long-term follow-up.

## Figures and Tables

**Table 1:** Baseline characteristics and demographics of our cohort.

	Study Cohort (n = 89)	Small Defects (n = 35)	Large Defects (n = 54)	*p*
female gender	50 (56%)	17 (49%)	33 (61%)	0.244
birth weight (g)	2924 (956–4180)	3124 (1500–4180)	2791 (956–4000)	0.009
length (cm)	51 (35–57)	51.5 (45–57)	50.0 (35–54)	0.005
gestational age (weeks)	37 + 3 (27 + 0–41 + 5)	38 + 0 (33 + 0–41 + 4)	37 + 1 (27 + 0–41 + 5)	0.003
ECMO	31 (35%)	1 (3%)	30 (56%)	<0.0001
ECMO days	7.6 (3–13)	5	7.6 (3–13)	
CDH repair with patch	54 (61%)	0 (0%)	54 (100%)	
recurrence	9 (10%)	-	9 (17%)	
median age at recurrence (months)	13 (12,183)	-	13 (12,183)	
time of follow-up (years)	8.2 (0.4–18.0)	6.9 (0.4–17.3)	9.1 (0.4–18.0)	0.04

Data are displayed as mean values with minimum and maximum values in brackets; age at recurrence is displayed as median values with interquartile range in brackets due to non-normal distribution of data; g, gram; cm, centimeter; ECMO, extracorporeal membrane oxygenation; CDH, congenital diaphragmatic hernia.

## Data Availability

The data presented in this study are available on request from the corresponding author.

## Supplementary Materials

The following supporting information can be downloaded at https://www.mdpi.com/article/10.3390/jcm13020620/s1: Table S1, “Geometric variables of the diaphragm in 89 left-sided CDH patients during long-term follow-up”; Table S2, “Geometric variables of the diaphragm in 89 left sided CDH patients with and without recurrence”; Table S3 “Geometric variables of the diaphragm in 57 left-sided CDH patients with and without scoliotic curves of the spine during long-term follow-up”; Table S4 “Geometric variables of the diaphragm in 36 left-sided CDH patients with and without a reduced thoracic area during long-term follow-up”.
