# Supplementary material for: Long-Term Evaluation of the Shape of the Reconstructed Diaphragm in Patients with Left-Sided Congenital Diaphragmatic Hernia Using Serial Chest Radiographs and Correlation to Further Complications
Source: J Clin Med. 2024 Jan 22;13(2):620. doi: 10.3390/jcm13020620 (PMC10816521; doi:10.3390/jcm13020620)
Supplement: Supplementary file 1 [file jcm-13-00620-s001.zip › jcm-2776508-supplementary.pdf]

		6 ± 2 months (19,9,10)	12 ± 2 months (22,13,10)	24 ± 4 months (23,18,10)	3.5 ± 0.5 years (20,7,10)	6 ± 1 year (34,20,10)	10 ± 2 years (27,9,10)	15.5 ± 2.5 years (19,2,10)
LDA (°)	large defects	28	28	28	29	28	30	32
	small defects	36	35	35	34	34	36	38
	controls	46	42	43	41	41	41	39
	large vs. small	*0.056*	***0.008***	***0.002***	*n.s.*	***0.001***	*0.075*	*n.s.*
	large vs. Controls	***<0.001***	***<0.001***	***<0.001***	***<0.001***	***<0.001***	***<0.001***	***0.005***
	small vs. Controls	*n.s.*	***0.035***	***0.004***	*n.s.*	***0.002***	*n.s.*	*n.s.*
RDA (°)	large defects	55	54	51	49	48	46	43
	small defects	53	47	47	49	44	44	37
	controls	46	44	45	42	42	42	40
	large vs. small	*n.s.*	***0.001***	*n.s.*	*n.s.*	***0.009***	*n.s.*	*n.s.*
	large vs. controls	***0.001***	***<0.001***	***0.008***	***0.007***	***0.001***	*n.s.*	*n.s.*
	small vs. controls	***0.038***	*n.s.*	*n.s.*	***0.034***	*n.s.*	*n.s.*	*n.s.*
LDD (mm)	large defects	54	61	67	75	76	87	100
	small defects	61	64	68	80	85	100	127
	controls	54	63	71	77	88	91	110
	large vs. small	***0.018***	*n.s.*	*n.s.*	*n.s.*	***0.001***	***0.004***	*n.s.*
	large vs. controls	*n.s.*	*n.s.*	*n.s.*	*n.s.*	***<0.001***	*n.s.*	*n.s.*
	small vs. controls	***0.032***	*n.s.*	*n.s.*	*n.s.*	*n.s.*	*n.s.*	*n.s.*
RDD (mm)	large defects	55	62	66	76	79	95	109
	small defects	59	67	68	77	89	97	136
	controls	58	64	75	78	95	95	114
	large vs. small	*n.s.*	*n.s.*	*n.s.*	*n.s.*	***<0.001***	*n.s.*	***0.029***
	large vs. controls	*n.s.*	*n.s.*	***<0.001***	*n.s.*	***<0.001***	*n.s.*	*n.s.*
	small vs. controls	*n.s.*	*n.s.*	***0.018***	*n.s.*	*n.s.*	*n.s.*	*n.s.*
LDH (mm)	large defects	12	11	11	12	12	13	12
	small defects	8	13	12	17	15	15	24
	controls	9	14	15	18	21	21	26
	large vs. small	***0.01***	*n.s.*	*n.s.*	***0.036***	*n.s.*	*n.s.*	***0.045***
	large vs. controls	*n.s.*	***0.026***	*n.s.*	***<0.001***	***<0.001***	***<0.001***	***<0.001***
	small vs. controls	*n.s.*	*n.s.*	*n.s.*	*n.s.*	***0.004***	***0.008***	*n.s.*
RDH (mm)	large defects	13	15	15	17	18	21	27
	small defects	10	15	14	15	18	19	35
	controls	10	14	14	18	22	23	27
	large vs. small	*n.s.*	*n.s.*	*n.s.*	*n.s.*	*n.s.*	*n.s.*	*n.s.*
	large vs. controls	***0.032***	*n.s.*	*n.s.*	*n.s.*	*n.s.*	*n.s.*	*n.s.*
	small vs. controls	*n.s.*	*n.s.*	*n.s.*	*n.s.*	*n.s.*	*n.s.*	*n.s.*
LDCI	large defects	5.1	6.3	7.3	6.7	7.4	7.2	11.4
	small defects	8.4	5.3	6.0	5.0	6.7	7.8	7.0
	controls	5.9	4.6	5.0	4.3	4.5	4.4	4.4

	large vs. small	***<0.001***	*n.s.*	*n.s.*	*n.s.*	*n.s.*	*n.s.*	*n.s.*
	large vs. controls	*n.s.*	***0.025***	***0.046***	***0.014***	***0.047***	***0.01***	*n.s.*
	small vs. controls	***0.006***	*n.s.*	*n.s.*	*n.s.*	*n.s.*	***0.011***	*n.s.*
RDCI	large defects	4.8	4.3	4.8	4.7	4.5	4.9	4.3
	small defects	6.2	4.6	5.0	5.3	5.3	5.3	4.2
	controls	6.1	4.8	5.3	4.5	4.6	4.2	4.3
	large vs. small	*n.s.*	*n.s.*	*n.s.*	*n.s.*	*n.s.*	*n.s.*	*n.s.*
	large vs. controls	*n.s.*	*n.s.*	*n.s.*	*n.s.*	*n.s.*	*n.s.*	*n.s.*
	small vs. controls	*n.s.*	*n.s.*	*n.s.*	*n.s.*	*n.s.*	*n.s.*	*n.s.*
LLLD (mm)	large defects	50	56	62	67	69	78	89
	small defects	54	60	65	74	80	91	125
	controls	51	61	67	75	84	88	108
	large vs. small	*n.s.*	*n.s.*	*n.s.*	*0.057*	***<0.001***	***0.007***	***0.001***
	large vs. controls	*n.s.*	*n.s.*	*n.s.*	***0.019***	***<0.001***	***0.027***	***0.002***
	small vs. controls	*n.s.*	*n.s.*	*n.s.*	*n.s.*	*n.s.*	*n.s.*	*n.s.*
RLLD (mm)	large defects	53	59	64	73	77	91	107
	small defects	55	62	65	75	85	96	134
	controls	55	59	70	74	87	91	109
	large vs. small	*n.s.*	*n.s.*	*n.s.*	*n.s.*	***0.002***	*n.s.*	***0.017***
	large vs. controls	*n.s.*	*n.s.*	***0.006***	*n.s.*	***0.001***	*n.s.*	*n.s.*
	small vs. controls	*n.s.*	*n.s.*	*n.s.*	*n.s.*	*n.s.*	*n.s.*	***0.036***
LTA (mm^2)	large defects	1,780	2,265	3,088	4,483	5,855	9,572	14,836
	small defects	1,990	2,805	3,241	5,312	7,552	12,294	24,534
	controls	1,830	2,236	3,008	4,531	7,084	8,388	15,464
	large vs. small	*n.s.*	*n.s.*	*n.s.*	*n.s.*	***<0.001***	*0.05*	***0.015***
	large vs. controls	*n.s.*	*n.s.*	*n.s.*	*n.s.*	***0.045***	*n.s.*	*n.s.*
	small vs. controls	*n.s.*	*n.s.*	*n.s.*	*n.s.*	*n.s.*	***0.015***	***0.031***
RTA (mm^2)	large defects	2,658	3,284	4,515	6,086	8,225	12,820	19,247
	small defects	2,712	3,663	4,681	6,267	9,174	13,075	24,228
	controls	2,297	3,236	3,806	5,828	8,350	10,051	17,531
	large vs. small	*n.s.*	*n.s.*	*n.s.*	*n.s.*	*n.s.*	*n.s.*	*n.s.*
	large vs. controls	*n.s.*	*n.s.*	*n.s.*	*n.s.*	*n.s.*	***0.034***	*n.s.*
	small vs. controls	*n.s.*	*n.s.*	*n.s.*	*n.s.*	*n.s.*	*n.s.*	*n.s.*

Table S1: Mean values of geometric variables of the diaphragm and thorax in 89 patients with left-sided congenital diaphragmatic hernia during a mean long-term follow-up of 8.2 years at 6 ± 2 months, 12 ± 2 months, 24 ± 4 months, 3.5 ± 0.5 years, 6 ± 1 year, 10 ± 2 years and 15.5 ± 2.5 years of age: left diaphragmatic angle (LDA), right diaphragmatic angle (RDA), left diaphragmatic diameter (LDD), right diaphragmatic diameter (RDD), left diaphragmatic height (LDH), right diaphragmatic height (RDH), left diaphragmatic curvature index (LDCI), right diaphragmatic curvature index (RDCI), left diaphragmatic curvature index (LDCI), left lower lung diameter (LLLD), right lower lung diameter (RLLD), left thoracic area (LTA), right thoracic area (RTA); numbers in brackets indicate the number of patients of which data was available at the respective time point during follow-up. Italic numbers indicate p-values; bold numbers indicate p values < 0.05; n.s., not significant; °, angle in degree; mm, millimeter; mm^2, square millimeter;

		LDA (°)	RDA (°)	LDD (mm)	RDD (mm)	LDH (mm)	RDH (mm)	LDCI	RDCI	LLLD (mm)	RLLD (mm)	LTA (mm^2)	RTA (mm^2)	TTA (mm^2)
	recurrence (3)	25	54	53	54	14	14	3.9	4.5	50	51	1,753	3,077	4,829
6 ± 2 months	no recurrence (25)	33	54	57	56	10	12	6.5	5.4	52	54	1,869	2,642	4,315
	p	n.s.	n.s.	n.s.	n.s.	n.s.	n.s.	**0.045**	n.s.	n.s.	n.s.	n.s.	n.s.	n.s.
	recurrence (5)	29	53	65	59	11	15	6.5	4.1	60	57	2,303	3,401	5,704
12 ± 2 months	no recurrence (30)	30	51	62	65	12	15	5.8	4.5	58	61	2,501	3,433	5,934
	p	n.s.	n.s.	n.s.	n.s.	n.s.	n.s.	n.s.	n.s.	n.s.	n.s.	n.s.	n.s.	n.s.
	recurrence (3)	25	55	70	66	12	19	6.5	3.6	64	62	3,006	4,721	7,728
24 ± 4 months	no recurrence (37)	32	49	67	67	12	14	6.5	5.0	63	64	3,185	4,573	7,758
	p	n.s.	n.s.	n.s.	n.s.	n.s.	**0.01**	n.s.	n.s.	n.s.	n.s.	n.s.	n.s.	n.s.
	recurrence (1)	28	53	71	74	10	19	7.1	3.9	60	68	2,741	3,932	6,673
3.5 ± 0.5 years	no recurrence (23)	30	49	76	77	14	17	6.2	4.8	69	74	4,713	6,132	10,845
	p	n.s.	n.s.	n.s.	n.s.	n.s.	n.s.	n.s.	n.s.	n.s.	n.s.	n.s.	n.s.	**0.037**
	recurrence (2)	28	52	72	70	9	17	8.4	4.0	67	66	3,669	6,065	9,734
6 ± 1 year	no recurrence (48)	31	46	80	83	13	18	7.0	4.9	73	81	6,593	8,636	15,050
	p	n.s.	n.s.	n.s.	**0.022**	**n.s.**	n.s.	**n.s.**	n.s.	n.s.	**0.004**	**0.009**	**0.018**	**0.015**
	recurrence (1)	30	52	83	83	12	15	7.2	5.7	73	83	8,364	13,175	21,538
10 ± 2 years	no recurrence (31)	32	45	90	95	13	20	7.3	5.1	81	92	10,103	12,684	22,787
	p	n.s.	n.s.	n.s.	n.s.	n.s.	n.s.	n.s.	n.s.	n.s.	n.s.	n.s.	n.s.	n.s.
	recurrence (3)	35	50	99	97	8	24	23	4.1	86	96	11,224	13,643	24,868
15.5 ± 2.5 years	no recurrence (16)	33	42	105	113	13	28	9.4	4.3	93	112	16,768	20,765	37,533
	p	n.s.	**0.013**	n.s.	n.s.	n.s.	n.s.	n.s.	n.s.	n.s.	n.s.	n.s.	**0.017**	**0.041**

Table S2: Mean values of geometric variables of the diaphragm and thorax in 89 patients with left-sided congenital diaphragmatic hernia with and without recurrence during a mean long-term follow-up of 8.2 years at 6 ± 2 months, 12 ± 2 months, 24 ± 4 months, 3.5 ± 0.5 years, 6 ± 1 year, 10 ± 2 years and 15.5 ± 2.5 years of age: left diaphragmatic angle (LDA), right diaphragmatic angle (RDA), left diaphragmatic diameter (LDD), right diaphragmatic diameter (RDD), left diaphragmatic height (LDH), right diaphragmatic height (RDH), left diaphragmatic curvature index (LDCI), right diaphragmatic curvature index (RDCI), left diaphragmatic curvature index (LDCI), left lower lung diameter (LLLD), right lower lung diameter (RLLD), left thoracic area (LTA), right thoracic area (RTA) and total thoracic area (TTA); numbers in brackets indicate the number of patients of which data was available at the respective time point during follow-up. Bold numbers indicate p values < 0.05; n.s., not significant; °, angle in degree; mm, millimeter; mm^2, square millimeter;

		LDA (°)	RDA (°)	LDD (mm)	RDD (mm)	LDH (mm)	RDH (mm)	LDCI	RDCI	LLLD (mm)	RLLD (mm)	LTA (mm²)	RTA (mm²)	TTA (mm²)
6 ± 2 months	scoliotic curve (6)	30	56	51	53	11	12	5.3	5.2	46	51	1,577	2,718	4,296
	no scoliotic curve (12)	35	53	59	57	9	10	7.4	5.8	54	54	2,013	2,693	4,706
	P	n.s.	n.s.	**0.011**	n.s.	n.s.	n.s.	n.s.	n.s.	**0.004**	n.s.	n.s.	n.s.	n.s.
12 ± 2 months	scoliotic curve (10)	31	53	61	60	10	14	6.5	4.5	56	58	2,047	3,296	5,343
	no scoliotic curve (15)	32	49	63	65	12	14	5.6	4.6	59	61	2,545	3,449	5,994
	P	n.s.	n.s.	n.s.	n.s.	n.s.	n.s.	n.s.	n.s.	n.s.	n.s.	n.s.	n.s.	n.s.
24 ± 4 months	scoliotic curve (8)	29	49	66	67	11	15	6.7	4.9	62	63	3,149	4,610	7,759
	no scoliotic curve (21)	32	48	68	67	12	14	6.6	4.8	64	65	3,132	4,627	7,759
	P	n.s.	n.s.	n.s.	n.s.	n.s.	n.s.	n.s.	n.s.	n.s.	n.s.	n.s.	n.s.	n.s.
3.5 ± 0.5 years	scoliotic curve (10)	32	49	75	77	11	18	7.9	4.8	66	72	4,405	6,240	10,645
	no scoliotic curve (8)	28	46	79	76	15	16	5.3	5	69	76	5,402	6,325	11,726
	P	n.s.	n.s.	n.s.	n.s.	**0.01**	n.s.	**0.045**	n.s.	n.s.	**0.049**	n.s.	n.s.	n.s.
6 ± 1 year	scoliotic curve (16)	30	49	79	81	13	18	6.7	4.8	72	79	5,696	8,032	13,226
	no scoliotic curve (29)	30	46	82	84	14	18	6.7	4.9	75	81	7,077	8,883	15,960
	P	n.s.	n.s.	n.s.	n.s.	n.s.	n.s.	n.s.	n.s.	n.s.	n.s.	**0.005**	n.s.	**0.004**

Table S3: Mean values of geometric variables of the diaphragm and thorax in 57 patients with left-sided congenital diaphragmatic hernia with and without scoliotic curves of the spine above the age of 8 years during early long-term follow-up at 6 ± 2 months, 12 ± 2 months, 24 ± 4 months, 3.5 ± 0.5 years and 6 ± 1 year: left diaphragmatic angle (LDA), right diaphragmatic angle (RDA), left diaphragmatic diameter (LDD), right diaphragmatic diameter (RDD), left diaphragmatic height (LDH), right diaphragmatic height (RDH), left diaphragmatic curvature index (LDCI), right diaphragmatic curvature index (RDCI), left lower lung diameter (LLLD), right lower lung diameter (RLLD), left thoracic area (LTA), right thoracic area (RTA) and total thoracic area (TTA); numbers in brackets indicate the number of patients of which data was available at the respective time point during follow-up. Bold numbers indicate p values < 0.05; n.s., not significant; °, angle in degree; mm, millimeter; mm², square millimeter;

		LDA (°)	RDA (°)	LDD (mm)	RDD (mm)	LDH (mm)	RDH (mm)	LDCI	RDCI	LLLD (mm)	RLLD (mm)
6 ± 2 months	reduced thoracic area (10)	33	56	53	49	8	11	7	4.7	49	48
	no reduced thoracic area (5)	35	53	58	61	11	11	5.5	6.2	53	57
	P	n.s.	n.s.	n.s.	**0.0026**	**0.0196**	n.s.	n.s.	n.s.	n.s.	**0.0039**
12 ± 2 months	reduced thoracic area (13)	28	59	62	59	10	14	6.5	4.2	57	56
	no reduced thoracic area (4)	30	50	64	63	12	14	6.0	4.7	58	59
	P	n.s.	**0.0142**	n.s.	n.s.	n.s.	n.s.	n.s.	n.s.	n.s.	n.s.
24 ± 4 months	reduced thoracic area (12)	25	52	62	63	11	15	6.1	4.3	58	60
	no reduced thoracic area (5)	32	48	68	68	12	14	6.5	5.1	63	66
	P	n.s.	n.s.	**0.038**	n.s.	n.s.	n.s.	n.s.	n.s.	**0.0428**	**0.0498**
3.5 ± 0.5 years	reduced thoracic area (13)	30	48	79	78	11	22	8.4	3.6	73	70
	no reduced thoracic area (2)	30	48	75	76	14	16	6.2	5.1	67	75
	P	n.s.	n.s.	n.s.	n.s.	n.s.	n.s.	n.s.	n.s.	n.s.	n.s.
6 ± 1 year	reduced thoracic area (19)	27	50	78	79	14	19	5.9	4.4	70	77
	no reduced thoracic area (7)	28	47	79	80	13	17	6.9	5.1	71	78
	P	n.s.	n.s.	n.s.	n.s.	n.s.	n.s.	n.s.	n.s.	n.s.	n.s.

Table S4: Mean values of geometric variables of the diaphragm and thorax during early long-term follow-up at 6 ± 2 months, 12 ± 2 months, 24 ± 4 months, 3.5 ± 0.5 years and 6 ± 1 year in 36 patients with left-sided congenital diaphragmatic hernia with a total thoracic area greater and smaller than the median total thoracic area (20,025mm^2) of the control group at the age of 10 ± 2 years: left diaphragmatic angle (LDA), right diaphragmatic angle (RDA), left diaphragmatic diameter (LDD), right diaphragmatic diameter (RDD), left diaphragmatic height (LDH), right diaphragmatic height (RDH), left diaphragmatic curvature index (LDCI), right diaphragmatic curvature index (RDCI), left lower lung diameter (LLLD) and right lower lung diameter (RLLD); numbers in brackets indicate the number of patients of which data was available at the respective time point during follow-up. Bold numbers indicate p values < 0.05; n.s., not significant; °, angle in degree; mm, millimeter; mm^2, square millimeter;
